# Analysis of *AVPR1A*, thermal and pressure pain thresholds, and stress in sickle cell disease

**DOI:** 10.3389/fpain.2022.1060245

**Published:** 2023-01-04

**Authors:** Keesha L. Powell-Roach, Yingwei Yao, Xueyuan Cao, Srikar Chamala, Margaret R. Wallace, Yenisel Cruz-Almeida, Robert E. Molokie, Zaijie Jim Wang, Diana J. Wilkie

**Affiliations:** ^1^ Department of Community and Population Health, University of Tennessee Health Science Center, College of Nursing, Memphis, TN, United States; ^2^ Department of Biobehavioral Nursing Science, University of Florida, College of Nursing, Gainesville, FL, United States; ^3^ Department of Pathology and Laboratory Medicine, Childrens Hospital of Los Angeles, Los Angeles, CA, United States; ^4^ Department of Molecular Genetics and Microbiology, University of Florida, College of Medicine, Gainesville, FL, United States; ^5^ University of Florida Genetics Institute, Gainesville, FL, United States; ^6^ College of Dentistry, University of Florida, Gainesville, FL, United States; ^7^ Pain Research and Intervention Center of Excellence (PRICE), University of Florida, Gainesville, FL, United States; ^8^ Department of Medicine, University of Illinois at Chicago, College of Medicine, Chicago, IL, United States; ^9^ Department of Pharmaceutical Sciences, Neurology and Bioengineering, University of Illinois College of Pharmacy, Chicago, IL, United States; ^10^ Medical Service, Jesse Brown VA Medical Center, Chicago, IL, United States

**Keywords:** sickle cell disease, pain, AVPR1A, genotype, quantitative sensory testing (QST), stress

## Abstract

**Aim:**

In patients with sickle cell disease (SCD), negative physical and emotional experiences result from intense chronic and acute pain episodes, but factors underlying these, and their interactions, are not well understood. The arginine vasopressin receptor 1a gene (*AVPR1A*) single nucleotide polymorphism rs10877969 has been previously associated with aspects of acute pain and stress related pain. In this study, we tested for associations between this SNP, thermal and pressure pain thresholds, clinical pain, and stress in people with SCD.

**Methods:**

150 adults enrolled with SCD completed pain intensity measures (Average Pain Intensity, API) and the Perceived Stress Questionnaire (PSQ). Thermal and pressure pain threshold data were available from quantitative sensory testing (QST), and rs10877969 genotypes were obtained.

**Results:**

In models adjusted for age and gender, between rs10877969 genotypes, we observed no significant differences in thermal (cold, *p* = 0.66; heat, *p* = 0.91) and mechanical (pressure, *p* = 0.33) pain thresholds. The association of rs10877969 with API (*p* = 0.09) was borderline, but non-significant with PSQ (*p* = 0.51). The correlation between clinical pain and environmental stress was significant, *r* = 0.18, *p* = 0.024, however, the interaction of genotype and PSQ was not significant (*p* = 0.63).

**Conclusion:**

Clinical and experimental pain were not significantly associated with the rs10877969 genotype. The rs10877969 genotype did not moderate the correlation between environmental stress and clinical pain in this population. However, a trend toward a protective T allele effect on average pain rating in SCD warrants future exploration of this SNP/gene in SCD.

## 1. Introduction

Biopsychosocial factors known to contribute to multiple pain conditions have not yet been studied in sickle cell disease (SCD), a genetic condition leading to microvasculature occlusion with resulting chronic and acute pain episodes (1–3). This absence of information on mechanisms that exacerbate the unpredictability of the potency and types of SCD-related pain is one of the major barriers impacting the ability to adequately address pain in this population. Pain results from multiple biological and psychological factors that have been investigated in other pain-related conditions. This includes genetic polymorphisms. The arginine vasopressin receptor (*AVPR1A*) single nucleotide polymorphism (SNP, rs10877969, C > T) was implicated in previous studies of pain not related to SCD. The purpose of this study was to determine whether pain and stress in adults with SCD is moderated by the rs10877969 genotype.

Pain phenotyping using quantitative sensory testing (QST) had been reported in many previous studies and has been shown to be safe for use in individuals with SCD with evidence of sensitization (4–7). However, no one has examined QST pain thresholds in association with the *AVPR1A* rs10877969 SNP in SCD.

In healthy individuals, previous work found a male-specific interaction of rs10877969 with stress, and observed that an endogenous analgesia mechanism can be activated by vasopressin if it has not already been activated by stress (8, 9). A moderate genetic (AVPR1A SNPs, including rs10877969) and psychological interaction was identified in studies of a model of exercise induced muscle injury (10). This SNP has also been found associated with autism and is theorized to affect gene expression because of its position in the *AVPR1A* promoter (11).

In patients living with SCD, harmful psychological effects from emotional stress have been shown to trigger or increase pain (12, 13). Recent studies have shown that activities such as relaxation and stress relieving modalities such as hypnosis (13), attention control with music, and guided relaxation in patients with SCD correlate significantly with reduced pain and stress (14–16). It has been shown in patients with SCD that anticipation of painful experiences enhances microvascular responses in blood flow, parasympathetic withdrawal, and sympathetic activation (13, 14, 17–19).

In our pilot study of the relationship between rs10877967 and self-reported pain in adults with SCD, we found that acute care utilization events and spontaneous reports of stress cited as an aggravator of pain were associated with this genotype (*p* = .04 and *p* = .002, respectively) (20). In particular, the CC genotype was associated with not spontaneously reporting stress as a pain aggravator (20). In this study, we extended those findings in a new sample of adults with SCD by examining QST pain thresholds and their psychosocial interactions on variables such as stress and pain. We hypothesized that thermal and mechanical pain thresholds would differ by rs10877969 genotype, and that it would influence the correlation between environmental stress and SCD clinical pain.

## 2. Methods and procedures

The dataset analyzed was generated from a SCD study whose detailed measures were published in 2020 (7, 21), briefly summarized here. Inclusion criteria: age ≥18 years; African ancestry; confirmed SCD diagnosis; SCD-related pain (≥3 on 1–10 scale) within previous 12 months; chronic SCD pain (>0 on scale 1–10 for at least half of the days over the previous 3 months); ability to speak and read standard English; ability to understand and sign the consent form. Exclusion criteria: legally blind; physically unable to complete the study measures; and/or had a confirmed diagnosis of diabetes mellitus or polyneuropathy. Patients were not excluded if they were using pain medications (opioid, non-opioid, or adjuvant).

Demographic data included: age, gender, ethnicity, race, marital status, education, income, partner, and type of sickle hemoglobin (HbS, HbC, or other). Participants provided blood samples and completed two questionnaires: PAINReportIt, providing information about recent and current pain [and its derivative Average Pain Intensity (API)] (22), and the Perceived Stress Questionnaire (PSQ) (23). In addition, using the left and right anterior forearm, somatosensory threshold data were obtained from Quantitative Sensory Testing (QST, thermal and mechanical pressure modalities). Methods were previously reported QST, body sites, and questionnaires (7, 21).

### 2.1. Measures

#### 2.1.1. Quantitative sensory testing

Quantitative sensory testing is a well validated battery of tests used at assess the function of the somatosensory system (24). Responses to sensations are tested and include, but are not limited to cold, hot, and pressure (25). QST was used to measure thermal and mechanical pain thresholds and was consistent with the European Federation of Neurological Sciences (EFNS) protocol (25). For the purposes of this study, the 30 × 30 mm thermode was placed on the skin and used to deliver standardized stimuli (26). The anterior forearm was used as the primary reference site (non-painful site). In the case where the anterior forearm was a painful, we selected the contralateral side for the non-painful site, if possible. The non-painful, reference site, was also used at the practice trial site (7).

Thermal pain thresholds (Hot and cold) were measured using the Medoc TSA -II sensory testing system. This is a computer-controlled device that generates and documents repeatable thermal stimuli that allows for assessment of sensory nerve function *via* pain thresholds, heat pain threshold (HPTh) and cold pain threshold (CPTh) (26, 27). The limits protocol was employed to avoid tissue damage. The cutoff temperature was 0°C for hold and 50°C for heat for all trials. The baseline temperature was 32°C. The temperature increased for heat and decreased for cold at a rate of 0.5°C per second until the participant pressed the button to indicate when pain was first felt. Thermal testing was stopped when the participant reported pain, at which time the participants verbally indicated the intensity of the pain they experienced. At that time, the participant was asked to assign the pain sensation a number on a 0–10 pain intensity scale.

Mechanical pain threshold was measured using standardized, calibrated von Frey filaments. Seven filaments were used and the sizes (thicknesses): 3.84 (0.6 g), 4.17 (1.4 g), 4.56 (4.0 g), 4.74 (6.0 g), 5.07 (26 g), and 5.88 (60 g), these were consistent with the EFNS protocol. The filament was perpendicularly touched to the skin until it obtained a s-shaped bend. The contact time to the skin was approximately 3 s. Each trial was approximately 2 inches from the previous trial up the forearm. With each trial, the filament thickness was increased. Testing was stopped when the participant reported feeling pain. At that time, the participant was asked to assign the pain sensation a number on a 0–10 pain intensity scale.

#### 2.1.2. PAIN*Report*It

PAIN*Report*It®, a valid and reliable self-reported pain assessment tool, is an internet-based version of the McGill Pain Questionnaire (MPQ) (28–30) and has been validated in patients living with SCD (31). This tool examines pain outcome measures with limited burden to the participant (32). The device is an interactive program that has a touch screen interface. It is used for pain assessment that may be self-administered by the patient or by a trained care provider or an assistant. The process takes approximately 15 min to complete. In addition to pain information, demographic variables are captured by the program (29). These variables included: diagnosis, age, gender, ethnicity, religion, education, occupation, income, substance use history, concurrent illnesses, 9-item medical history checklist related to SCD, and items examining previous pain experiences, which include worst toothache, headache and stomachache. Recalling worst pain experiences allows the participant recall the magnitude of common pain experiences and compare them to pain from the QST testing.

#### 2.1.3. Average pain intensity

Average Pain intensity (API) is taken as the mean of PAIN*Report*It's three 0 to 10 Pain Intensity Number Scales (PINS) (33, 34): (1) current pain, (2) least in the past 24 h, and (3) worst pain in the past 24 h. The PINS measured the patient's perception of the level of pain now (34) and it provides ratio level data (31, 33, 34). The patient designates the pain as a number between 0 and 10, where 0 is “no pain” and 10 is “pain as bad as it could be” (34, 35). Concurrent (*r* = 0.80 to 0.89) and construct validity (34, 36) had been reported for this tool and standardized instructions (33, 34) are available.

#### 2.1.4. Perceived stress questionnaire

The Perceived Stress Questionnaire (PSQ) is a validated 30-question instrument used to assess stressful life events (37). This tool takes about 15 min to complete. Internal consistency (*α* = 0.90 to 0.92) and test re-test reliability (*r* = 0.82) have been evaluated for this scale (37). This scale evaluates how frequently the participant experiences stress-related feelings (37). Responses range from “almost never” (1) to “usually” (4). Higher scores represent greater stress (37). The PSQ Index is generated by (raw score-30)/90, resulting in final scores ranging from 0 to 1 (37).

Participant leukocyte DNA genotype data were generated using the 800,000-SNP Axiom Precision Medicine Research Array. Because the SNP of interest, rs10877969, is not on the PMRA array, we imputed this genotype with high confidence using the Michigan Imputation Server (https://imputationserver.sph.umich.edu) with imputation score *R*^2^ > 0.8. Under an additive genetic model with a sample size of 150, we have 84% power to detect a significant association between SNP and pain score(s) with effect size of 0.06 defined by Cohen (1988) at alpha 0.05 level (38).

We used the statistical package R to interrogate the association of demographic variables and SNP genotype with thermal and pressure pain thresholds, clinical pain, and environmental stress. The rs10877969 genotype was coded based on the number of C alleles based on previous reports (e.g., CC = 2, CT = 1, TT = 0). To better represent the data by normal distribution, we log transformed thermal and pressure pain thresholds, clinical pain, and environmental pain variables. We used robust linear regression to attenuate the impact of model fitting by high influential points. The statistical significance cut-off was *p* < 0.05.

## 3. Results

A total of 186 evaluable SCD patients met inclusion criteria for the parent study, and of those, 150 participants completed the PSQ. The participants were mainly Black or African American (97.3%), single (70%), with some college or high school education (87%), income ≤30 K (77%), age 18–39 (66.7%) and female (62.7%). The majority of participants had SS Hgb type (70.7%), with others being heterozygous with hemoglobin S and C (SC Hgb). Demographics along with sickle cell types are also provided in Table 1. In this cohort, the rs10877969 genotype frequency distributions were: CC *n* = 43 (28%), CT *n* = 67 (44%), TT *n* = 40 (26%) (Table 1). The analytic sample included 150 participants with completed genotype and PSQ data and 148 participants from whom we also had QST data.

**Table 1:** Demographics and outcome measurements (*N* = 150).

Demographics	Levels	*N* (%) Median (IQR)
Age		33.5 (26.25–44)
Age group	18–39	100 (66.67%)
	≥40	50 (33.33%)
Gender	Female	94 (62.67%)
	Male	56 (37.33%)
Ethnicity	Hispanic/Latino	4 (2.67%)
	Not Hispanic/Latino	146 (97.33%)
Race	Black/African-American	146 (97.33%)
	Multi-race/Multi-ethnic	3 (2%)
	Other	1 (0.67%)
Marital status	Divorced/Separated	5 (3.33%)
	Married/Partnered	34 (22.67%)
	Single	105 (70%)
	Widowed	3 (2%)
	Missing	3 (2%)
Education	HS^−^	63 (42%)
	SC	68 (45.33%)
	BS^+^	17 (11.33%)
	Missing	2 (1.33%)
Income	<10 K	55 (36.67%)
	10 K–30 K	61 (40.67%)
	>30 K	27 (18%)
	Missing	7 (4.67%)
Partner	No	113 (75.33%)
	Yes	34 (22.67%)
	Missing	3 (2%)
Sickle Hgb Type	SC	30 (20%)
	SS	106 (70.67%)
	Other	11 (7.33%)
	Missing	3 (2%)
rs10877969	CC	43 (28.67%)
	TC	67 (44.67%)
	TT	40 (26.67%)
	Missing	0 (0%)

HS^−^, High school; SC, Some college; BS^+^, Bachelor's degree.

### 3.1. Rs10877969 association with thermal pain thresholds

We examined the association of demographic variables and rs10877969 SNP genotype with thermal pain thresholds. The mean, standard deviation, max and min for thermal pain thresholds for the 148 participants are as follows: cold pain threshold 25.38 (5.72), [0–30.91], heat pain threshold 38.53 (4.00), [33.24–47.63] (Table 2). Robust linear regressions adjusting for age and gender indicated that neither cold nor heat pain thresholds were significantly associated with rs10877969 genotype (*p* = 0.66, and *p* = 0.91, respectively) (Table 3).

**Table 2:** Mean and standard deviation of variables with rs10877969 genotype.

Var	Genotype	N	Mean Std	Median (IQR)	Min-Max
API	All	150	4.39 ± 2.43	4.67 (2.67, 6)	0, 9.33
	CC	43	4.76 ± 2.59	5 (2.83, 6.5)	0, 9.33
	TC	67	4.48 ± 2.38	4.67 (2.58, 6)	0, 9.33
	TT	40	3.85 ± 2.28	4.33 (2.71, 5.33)	0, 9
Cold Pain Threshold^a^	All	148	25.38 ± 5.72	27.56 (24.09, 28.92)	0, 30.91
	CC	42	25.67 ± 4.74	27.33 (24.47, 28.66)	9.92, 30.77
	TC	66	25.31 ± 5.76	27.48 (24.01, 29)	8.4, 30.91
	TT	40	25.2 ± 6.66	27.86 (23.78, 29.22)	0, 30.75
Heat Pain Threshold^a^	All	148	38.53 ± 4	37.13 (35.22, 41.66)	33.24, 47.63
	CC	42	38.27 ± 3.74	37.41 (35.04, 41.61)	33.24, 46.3
	TC	66	38.8 ± 3.95	37.42 (35.45, 41.2)	33.75, 47.63
	TT	40	38.37 ± 4.42	36.61 (34.69, 42.26)	33.67, 47.37
Pressure Pain Threshold^b^	All	148	11.87 ± 18.69	4 (1.4, 10)	0.6, 60
	CC	42	8.24 ± 13.75	4 (1.4, 6)	0.6, 60
	TC	66	12.89 ± 20.1	4 (1.4, 10)	0.6, 60
	TT	40	14.01 ± 20.6	4 (1.4, 10)	0.6, 60
PSQ	All	150	0.37 ± 0.17	0.34 (0.26, 0.48)	0.06, 0.92
	CC	43	0.37 ± 0.17	0.38 (0.24, 0.43)	0.06, 0.84
	TC	67	0.4 ± 0.18	0.34 (0.26, 0.52)	0.11, 0.92
	TT	40	0.34 ± 0.16	0.31 (0.23, 0.46)	0.08, 0.7

^a^ Thermal pain thresholds are measured in degrees Celsius (°C).

^b^ Pressure pain thresholds are measure in grams of force.

**Table 3:** Experimental and clinical pain associations with rs10877969 adjusting for relevant variables.

Dependent Variable	Independent Variable	Coef	CI 95	*p-*value
Cold Pain Threshold				
	rs10877969	−0.170	−0.916–0.577	0.6556
	Sex (Male)	−0.438	−1.591–0.714	0.4567
	age	0.001	−0.049–0.05	0.9721
Heat Pain Threshold				
	rs10877969	0.053	−0.86–0.967	0.909
	Sex (Male)	0.512	−0.898–1.922	0.479
	age	−0.005	−0.066–0.055	0.863
Pressure Pain Threshold				
	rs10877969	0.883	0.69–1.131	0.325
	Sex (Male)	1.298	0.885–1.903	0.183
	age	0.998	0.982–1.014	0.798
API				
	rs10877969	0.485	−0.072–1.041	0.090
	Sex (Male)	−0.545	−1.402–0.312	0.213
	age	0.019	−0.017–0.056	0.303
PSQ				
	rs10877969	0.012	−0.025–0.049	0.511
	Sex (Male)	0.008	−0.049–0.065	0.782
	age	−0.002	−0.005–0	0.083
API (Interaction)				
	rs10877969	0.780	−0.605–2.165	0.270
	PSQ	3.170	−1.293–7.634	0.162
	rs10877969:PSQ	−0.886	−4.467–2.694	0.628
API/PSQ (correlation)				
		Corr 0.18		0.024

### 3.2. Rs10877969 association with mechanical pain thresholds

The mean, standard deviation, max and min for mechanical pain thresholds are as follows: *N* = 148, 11.87 (18.69), [0.6–60] (Table 2). In robust linear regression models adjusted for age and gender, we observed no significant association between mechanical pain thresholds and rs10877969 genotype (*p* = 0.33) (Table 3).

### 3.3. Rs10877969 association with clinical pain and environmental stress

The mean, standard deviation, max and min for clinical pain and environmental stress in the full cohort of 150 are as follows: clinical pain, 4.39 (2.43), [0–9.33] and environmental stress, 0.37(0.17), [0.06–0.92] (Table 2). The Median (Inter Quartile Range, IQR) for API was: CC 5 (2.83, 6.5), CT 4.67 (2.58, 6), TT 4.33 (2.71, 5.33); and PSQ: CC 0.38 (0.24, 0.43), CT 0.34 (0.26, 0.52), TT 0.31 (0.23, 0.46) (Table 2). In robust linear regression models adjusting for age and sex, environmental stress (coef = 0.012; *p* = 0.51) was not significantly associated with rs10877969 (Table 3). However, there was a trend toward association of clinical pain (API) and genotype (coef 0.485, *p* = 0.09) (Table 3). We fitted a robust linear regression model of API with rs10877969, PSQ and the interaction term. We did not observe significant interaction of rs10877969 and PSQ in association with API (−0.886, *p* = 0.63) (Table 3). However, the correlation between clinical pain and environmental stress was significant, *r* = 0.18, *p* = 0.024.

Additionally, an exploratory analysis was done examining a male specific interaction. In our sample we examined the interaction of sex, SNP genotype and PSQ; sex, SNP genotype and API. No significant interactions were found.

## 4. Discussion

In this analysis of data from 150 adults with SCD, we evaluated association of clinical and experimental pain and environmental stress with the rs10877969 SNP of the *AVPR1A* gene. We found that experimental pain as indicated by thermal and mechanical pain thresholds, clinical pain, and environmental stress were not significantly associated with the rs10877969 genotype, although the *p-*value for genotype vs. API was 0.09. The rs10877969 SNP did not moderate the association between clinical pain (API) and stress (PSQ) in this population.

There have been variable conclusions in reports of stress related pain in patients with SCD, such as pain resulting from the stress of perceived injustice (23), and mental stress being associated with vasoconstrictions in patients with SCD and healthy controls (19). Here we found a correlation between clinical pain (API) and the PSQ measure of stress in SCD. We previously reported that individuals with SCD and rs10877969 CC genotype were less like to cite stress as aggravating their pain compared to the other two genotypes (20). Utilization and composite pain index were proxies for acute pain and chronic pain (respectively). We did not measure API in the previous study. CT was higher for acute pain (6.1); (CC = 3.9, TT 3.3). The genotype level was measured in the previous study, and we did not control for age and sex. In the current study CC is higher for API, and allele level was measured (TT = 0, CT = 1, CC = 2). Chronic clinical pain is associated with the CC allele whereas a proxy for acute pain is associated with the CT allele, and TT has the lowest score in both samples.Currently, there is inconclusive evidence for the contribution of the rs10877969 CC genotype to average pain intensity. The positive association between C allele and API, though statistically not significant in this sample, warrants further study. The current findings in environmental stress are in contrast to that of our previous study (20). It is important to note, however, that the PSQ is a different measure than spontaneously citing stress as a pain aggravator. In our pilot study we examined the relationship between the SNP, self-reported pain, stress, and acute pain. In both studies, the C allele is associated with more pain. Previous studies showed that rs10877969 was related to indicators of environmental stress and acute pain (8, 9, 20), however, our findings in this cohort differed from those other studies. Individuals in this study appeared not to be very stressed. PSQ queries stressful feelings over the past month (37) on a 0 to 1 scale. At the twenty-fifth percentile, the average stress score was 0.26 and at the seventy-fifty percentile, the average stress score was 0.48. It is also possible that a different stress scale may be better suited for this population whose disease process is variable. We found that rs10877969 did not influence the weak but significant association between pain and stress in this sample of patients with SCD.

### 4.1. Study limitations

Since reports of stress are not consistently associated with the rs10877969 genotype, the precise measurement tool may be important to properly interrogate this relationship. Thus, PSQ may not be the appropriate tool to measure stress in this population or investigators may need to sample in ways to include individuals with higher stress levels in future studies. Additionally, use of antidepressants was not an exclusion criterion in this study. We cannot rule out if PSQ scores were impacted. Lastly, a larger sample size testing multiple measurements is needed for future studies, to fully answer the question about this SNP's role in SCD.

## 5. Conclusion

In this study of *AVPR1A* rs1087796 in a cohort of individuals with SCD, we examined a direct measure of stress and measures of experimental and clinical pain. Although none of the associations were significant, the trend toward a protective T allele effect on pain in SCD warrants future exploration of this SNP/gene in SCD, perhaps by analyzing more individuals, perhaps starting with the ends of the SCD pain spectrum. As this SNP is located in the promoter region of the gene, further research is warranted to examine the functional impact of this polymorphism on gene transcription and protein production, to understand underlying biological impact. Analysis of additional AVPR1A SNPs, particularly in linkage disequilibrium with rs10877969, in a larger SCD cohort, could also shed light on the involvement of this gene in SCD pain.

## Data Availability

The raw data supporting the conclusions of this article will be made available by the authors, without undue reservation.
